# PROJECTION OF MEDICAID SPENDING ON PEOPLE WITH ALZHEIMER’S DISEASE AND OTHER RELATED DEMENTIAS

**DOI:** 10.1093/geroni/igad104.2831

**Published:** 2023-12-21

**Authors:** Howard Degenholtz

**Affiliations:** University of Pittsburgh, Pittsburgh, Pennsylvania, United States

## Abstract

As the US population is aging, the number of people with Alzheimer’s Disease and Other Related Dementias (ADRD) is increasing. People with ADRD have high expenditures on medical care and long-term services and supports (LTSS). Medicaid is the largest single payor for LTSS, however, the literature on the demographic trends and epidemiology of people with ADRD does not offer projections of Medicaid spending. The goal of this study was to develop an estimate for future Medicaid spending for people with ADRD for the state of Pennsylvania. The approach taken was to develop a demographic projection of the size of the older population, then estimate the fraction of people with ADRD who are eligible for Medicaid. Next, we used estimates of current spending trends to project future spending through 2025. Nearly all Medicaid participants with ADRD are dually eligible for both Medicaid and Medicare, however, Medicare managed care encounter data were not available for analysis. Thus, the prevalence of ADRD was constructed using Medicaid and Medicare fee-for-service (FFS) claims data. Two alternate estimates of the prevalence of ADRD among Medicare managed care enrollees were used (same as Medicare FFS, or slightly lower). Estimates were generated under several growth rates in per person spending on home and community-based services (HCBS). The results shows that growth in HCBS spending is the largest factor in predicting spending, leading to estimates ranging from $2.8 billion to $3.5 billion. Future research will extend the model to additional years.

